# Drawing the inner world: exploring autistic perception through virtual reality art-making

**DOI:** 10.3389/fpsyg.2026.1739991

**Published:** 2026-05-01

**Authors:** Sarune Savickaite, Rebecca Airlie, Neil McDonnell, David Simmons

**Affiliations:** 1 Department of Psychology, University of Exeter, Exeter, United Kingdom; 2 School of Psychology and Neuroscience, University of Glasgow, Glasgow, United Kingdom; 3 School of Philosophy, University of Glasgow, Glasgow, United Kingdom

**Keywords:** autism, drawing, neurodiversity, perception, sensory experience, virtual reality

## Abstract

First-person accounts of perceptual experience in autism remain underrepresented in psychological research. This exploratory study examined how autistic adults use immersive media to externalize and reflect on perception. Six adults with a formal autism diagnosis (four male, two female; mean age 22.7 years) completed a virtual reality (VR) protocol that combined free drawing in the VR tool Open Brush with the Think Aloud method and a brief interview. To contextualize individual sensory and cognitive profiles, participants completed the Autism Quotient (AQ) and the Glasgow Sensory Questionnaire (GSQ). Reflexive thematic analysis of verbal reports, interviews and digital drawings identified three overarching themes: immersion, artistic expression and individuality, and escapism. Participants described VR as calming, engaging and controllable, with the ability to explore artworks from multiple perspectives and to adjust the environment to reduce sensory demands. Creative approaches were highly individual ranging from detailed figurative drawings to abstract compositions. These often reflected personal interest and preferred sensory qualities. Quantitative scores illustrate the heterogeneity in attention to detail and sensory sensitivity, supporting the qualitative emphasis on variability within autism. The findings demonstrate the feasibility and value of VR drawing as a neurodiversity- affirming participatory method, with potential applications in therapy and in communicating autistic perceptual experience to non-autistic audiences.

## Introduction

Our understanding of the world is shaped by the ways our visual system organizes incoming sensory data into coherent and meaningful experiences. This process, known as perceptual organization, allows us to extract both the fine details and the overall structure of our environment (Wagemans et al., 2012; Goldstein and Brockmole, 2016). While such mechanisms are broadly shared, *how* we perceive and interpret this information can differ significantly between individuals.

These differences in perceptual processing are shaped by a range of factors including developmental history, mood, gender, and neurotype (Freeseman et al., 1993; Sadler-Smith, 2011; Simmons and Todorova, 2018). In autistic individuals, perceptual differences are well-documented and often emphasize enhanced sensitivity to detail, unique sensory experiences, and differing responses to visual stimuli (Simmons et al., 2009).

Autism is a neurodevelopmental condition characterized by persistent difficulties in social communication and interaction, alongside restricted, repetitive patterns of behavior, interests, or activities (American Psychiatric Association [APA], 2022). Autism is a spectrum condition, meaning it affects individuals in different ways and to varying degrees. While some autistic individuals may have co-occurring intellectual disabilities or require substantial support in daily life, others may be highly independent and skilled in particular areas. As noted by National Autistic Society (2024), many autistic people experience sensory sensitivities, differences in communication styles, and a strong preference for routine.

When discussing autism, it is essential to be respectful and inclusive in language use. The National Autistic Society (2024) highlights that many autistic people prefer identity-first language (e.g., “autistic person”) rather than person-first language (e.g., “person with autism”), as it reflects autism as an integral part of their identity. As a result, we will use identity-first language throughout this text.

Autistic people frequently describe their sensory world in ways that challenge typical assumptions. Autobiographical accounts speak vividly of sensory overload, fragmented vision, and intense perceptual focus-experiences that remain largely inaccessible to traditional experimental methods (Grandin, 2006; Higashida, 2013; Robertson and Simmons, 2015). For example, Temple Grandin’s description of “thinking in pictures” or Donna Williams’ portrayal of light particles enveloping her vision capture a world that is rarely represented in clinical literature (Grandin, 2006; Williams, 1992).

Despite growing awareness, autism research has historically prioritized behavioral symptoms over perceptual experience (Robertson and Baron-Cohen, 2017), often neglecting the internal, first-person accounts that are central to understanding the condition. This mismatch between the measurable and the felt has resulted in a gap between lived experience and empirical research—a gap that participatory, arts-based, and person-centered research is now seeking to bridge (Happé and Frith, 2020).

As researchers increasingly adopt neurodiversity-affirming frameworks, there is renewed interest in methods that empower autistic individuals to communicate their experiences in non-verbal, creative, and self-led ways. Drawing, for instance, has long been used in therapeutic settings to support expression and reflection (Oster and Crone, 2004; Guillemin, 2004), and it offers a promising avenue for capturing perceptual differences that might otherwise remain invisible.

In parallel, Virtual Reality (VR) is emerging as a powerful tool for psychological research, offering immersive, interactive environments that feel less socially demanding and more ecologically valid—particularly for autistic participants (Maskey et al., 2014). VR can enable individuals to express and share aspects of perception through embodied movement and creative interaction. Combined with drawing tasks and Think Aloud protocols (Arsal and Eccles, 2017)—where participants narrate their thoughts during a task—VR has the potential to reveal not only *what* is experienced, but *how* it is processed in the moment.

Early applications of VR in autism research focused primarily on social and behavioral outcomes, including social skills training, emotion recognition, and exposure to anxiety-provoking situations such as public transport or crowded environments (Maskey et al., 2014; Lorenzo et al., 2019). These studies demonstrated that VR can reduce anxiety and support learning by allowing graded exposure and repeatable practice within safe, predictable settings. However, this line of work has largely framed VR as a remediation tool, aimed at helping autistic individuals adapt to neurotypical social environments, rather than as a medium for exploring autistic experience in its own right.

Beyond behavioral training, VR has also been used to investigate perceptual and attentional styles associated with autism and related neurodivergent traits. Studies using immersive environments have shown that VR can reveal differences in visual processing, spatial attention, and sensory integration that are not easily captured by conventional 2D tasks (Savickaite et al., 2021). Importantly, VR allows perception to be studied not only as a response to stimuli but as an active, embodied process that unfolds through movement, exploration, and interaction with space.

More recent work has begun to recognize VR as a participatory and expressive medium that can support self-directed exploration and communication. In particular, immersive environments give users control over sensory parameters such as lighting, color, movement, and sound, which is especially important given the prevalence of sensory sensitivities in autism (MacLennan et al., 2021). The ability to regulate stimulation and perspective may reduce cognitive and sensory load, enabling autistic participants to engage more comfortably and for longer periods than in physical or socially demanding settings.

## The current study

In this study, we aimed to explore the perceptual experiences of autistic individuals using a combination of VR drawing tasks and the Think Aloud method. We were particularly interested in capturing self-led expressions of visual processing, exploring how participants interact with immersive environments and externalize their inner perceptual world. Drawing from the neurodiversity paradigm and person-centered research models, we sought to move beyond traditional psychometric approaches to engage participants in a more meaningful and reflective exploration of their sensory experiences. Through this mostly qualitative approach, we aimed to contribute new insights into the rich and varied ways autistic individuals perceive, process, and communicate their world.

## Materials and methods

### Participants

This study was approved by the University of Glasgow. Recruitment took place via social media platforms, including the University of Glasgow mailing lists. Participants received either £6 or course credit for taking part.

Data collection occurred between January and February 2022 in the Virtual Reality (VR) lab at the University of Glasgow. The sample consisted of six participants (four male, two female), with a mean age of 22.7 years (SD = 3.4). All participants self-reported having previously received a formal autism diagnosis. Diagnostic documentation was not requested and diagnoses were not independently verified. This approach is common in qualitative autism research that recruits adults through community or online networks, where self-reported diagnosis is typically accepted to reduce barriers to participation and respect participants’ autonomy (e.g., Cage et al., 2018; Botha et al., 2021; Nicolaidis et al., 2019). In line with neurodiversity-affirming and participatory research principles, the study prioritized participants’ lived experiences rather than clinical verification of diagnosis.

Participants were asked about visual impairments and previous experience with VR. Three participants (50%) reported wearing spectacles, though none had significant vision impairments. Two participants (33%) had prior experience using VR, including one who owned an Oculus Quest headset. Demographic details are presented in Table 1.

**TABLE 1:** Demographic information of the participants.

Participant	Age	Gender
Participant 1	19	Male
Participant 2	20	Male
Participant 3	26	Female
Participant 4	27	Male
Participant 5	20	Female
Participant 6	24	Male

### Materials

Participants used an Nvidia GTX 1080 I Gaming PC (Windows 10) and an HTC Vive Pro headset with two controllers. TheBlu (WEVR, 2016) was used to introduce participants to the VR environment, and Open Brush (Icosa Gallery, 2021) was used for the drawing task. The experiment was run using SteamVR software (https://store.steampowered.com/).

### TheBlu

To familiarize themselves with VR, participants spent approximately 10 min immersed in *theBlu*, an underwater VR experience featuring high-quality visuals, ambient audio, and limited interactivity. This was intended to introduce the immersive qualities of VR.

### Open Brush

Open Brush software enabled participants to create three-dimensional drawings using a variety of brushes, colors, and backgrounds (e.g., night sky, space, snow). While the environment had no direct effect on drawings, lighting variations affected how brush colors appeared. Common tools included the *eraser* (to remove strokes) and *teleport*, which allowed movement and different perspectives within the drawing space.

### Questionnaires

#### Technical proficiency questionnaire

The Technical Proficiency Questionnaire (TPQ) Included nine items assessing experience with VR, general tech use, and visual impairments. The questionnaire was administered via Microsoft Office Forms. The full questionnaire is available at https://osf.io/6k9cw/overview.

#### Autism spectrum quotient

The Autism Spectrum Quotient Questionnaire (AQ) assesses autistic traits across five domains (social skills, attention switching, attention to detail, communication, imagination) using a 50-item, four-point Likert scale. AQ has demonstrated acceptable internal consistency and test–retest reliability, with several studies supporting its utility in identifying traits associated with autism in both clinical and non-clinical populations (Ruzich et al., 2015; Hoekstra et al., 2008); however, concerns have been raised regarding the validity of its total score and the coherence of its subscales, suggesting the need for cautious interpretation and use primarily for research rather than diagnostic purposes (English et al., 2020). At the time the study was conducted (2022), the AQ was widely used as a research screening and trait-profiling tool; however, it was not used here for diagnostic purposes, and its scores are interpreted only descriptively.

#### Glasgow Sensory Questionnaire

The Glasgow Sensory Questionnaire (GSQ) is a 42-item questionnaire assessing hyper- and hypo-sensitivities across seven sensory domains (visual, auditory, gustatory, olfactory, tactile, vestibular, proprioceptive). Responses are rated on a 0–4 scale (Never to Always). Scores range from 0 to 168, with higher scores indicating more pronounced sensitivities. The GSQ has strong reliability and cross-cultural validity (Horder et al., 2014; Takayama et al., 2014; Sapey-Triomphe et al., 2018) and correlates positively with AQ scores (Robertson and Simmons, 2013; Ward et al., 2017).

### Procedure

Before the study, participants provided informed consent and completed the AQ, GSQ, and TPQ. They were then introduced to the VR equipment and allowed to ask any clarifying questions.

*TheBlu* (WEVR, 2016) was used to help participants acclimatize to VR. Participants had ample physical space to move safely. For those uncomfortable with underwater scenes, *Google Earth VR* was offered, though all participants chose *theBlu*. Each spent 5–10 min familiarizing themselves with VR, with scheduled breaks to reduce the risk of disorientation (Sousa Santos et al., 2009). Cybersickness was effectively mitigated by limiting exposure time and incorporating regular breaks during virtual reality sessions, which helped reduce sensory conflict and motion-related discomfort (Rebenitsch and Owen, 2016).

Next, participants were introduced to *Open Brush*. They explored brush types, colors, and environments, and were encouraged to draw freely. The experimenter prompted reflection by asking participants to explain their choices. Where needed, drawing prompts (e.g., house, tree, person) adapted from art therapy studies were provided (Chung et al., 2020; Kaimal et al., 2020). The full list of prompts is available at https://osf.io/gy6vj/.

As light and environment changes can cause discomfort for autistic individuals (Robertson and Simmons, 2015), each session was capped at 1 h, with breaks encouraged.

After the drawing task, participants were invited to complete a semi-structured interview based on previous research into autism diagnosis, perceptual experiences, and communication with neurotypical people (Russell et al., 2019; Thompson-Hodgetts et al., 2020). Interview questions included:

1. What is your diagnosis journey? Could you tell us anything more about it?
2. What could you share about your perceptual world that would help us understand it better?
3. Are you artistic? Does creativity help you express your perceptual experiences?
4. Do your perceptual experiences change? Do you think they differ from those of other autistic individuals?
5. How did you feel during the experiment? Any suggestions for improvement?
6. Do you think VR could help others understand autistic perception?

If participants were uncomfortable answering any questions, they were skipped. In many cases, participants spontaneously addressed these topics during the drawing task and were later asked to elaborate. All drawing sessions were recorded (video and audio), transcribed, and anonymized.

### Data analysis

#### Quantitative data

AQ and GSQ scores were analyzed using RStudio Team (2021). Total and subscale scores were calculated for each participant to support the qualitative findings.

#### Qualitative data

Audio recordings were transcribed using *Descript* (Version 33.1.1). SteamVR screen recordings and screenshots were also analyzed. Coding and thematic analysis were conducted in NVivo 12 (QSR International, 2018).

All qualitative data-including think-aloud content during drawing and interview responses-were analyzed using reflexive thematic analysis (Braun and Clarke, 2006). This six-step process involved familiarization, initial coding, theme development, theme review, naming and definition, and producing the report. Due to the small sample size, results were presented on a case-by-case basis. Two researchers independently analyzed the data and collaboratively finalized the coding and themes.

## Results

### Quantitative data

The total AQ and GSQ scores were calculated for each participant. Subscale scores were also assessed, and the following tables include the results. The AQ scores are presented in Table 2. For each participant, total GSQ score, GSQ hypersensitivity score, GSQ hyposensitivity score and seven modalities were calculated and shown in Tables 3–5.

**TABLE 2:** AQ subscales and full score for each participant.

Participants	AQ subscales					AQ
	Social skills	Attention switching	Attention to detail	Communication	Imagination	
1	4	7	5	4	4	24
2	7	10	10	10	10	47
3	3	1	4	2	2	12
4	3	6	5	7	7	28
5	4	4	3	6	6	25
6	2	10	7	7	7	33

AQ subscale and total scores for each participant. Scores are presented for descriptive purposes only to illustrate heterogeneity within the sample (Woodbury-Smith et al., 2005).

**TABLE 3:** GSQ scores for individual participants with subscales.

Participants	GSQ subscales		GSQ
	Hypersensitivity	Hyposensitivity	
1	21	23	44
2	68	52	120
3	35	28	63
4	26.8	19	45.8
5	37	33	70
6	34	12	46

Where, for example, over 50% of population scores above 97 for the full GSQ score (Millington et al., 2022).

**TABLE 4:** GSQ Hypersensitivity subscales scores for individual participants.

Participants	Hypersensitivity						
	Visual	Tactile	Auditory	Olfactory	Gustatory	Proprioception	Vestibular
1	5	4	5	0	1	1	5
2	11	11	10	10	7	9	10
3	5	4	7	6	7	1	5
4	5	6	2	2	0	5	6.8
5	6	7	4	6	3	6	5
6	2	4	8	6	5	4	5

**TABLE 5:** GSQ Hyposensitivity subscales scores for individual participants.

Participants	Hyposensitivity						
	Visual	Tactile	Auditory	Olfactory	Gustatory	Proprioception	Vestibular
1	5	2	3	3	3	2	5
2	8	4	12	11	4	6	7
3	2	6	7	3	5	3	2
4	2	0	3	5	2	4	3
5	5	4	8	6	3	3	4
6	0	0	4	4	2	2	0

It is notable that only participant 2 scored high on the AQ. Participants 4 and 6 are only just above the 26 cut-off point (Woodbury-Smith et al., 2005) and the remaining participants are close or even below general population means. The AQ scores illustrate substantial heterogeneity in self-reported autistic traits within the sample. Given the small sample size and known psychometric limitations of the AQ, particularly in diagnosed adults and those with co-occurring conditions, these scores are reported only as contextual descriptors and are not interpreted as indicators of diagnostic validity or severity (Baron-Cohen et al., 2001; Ashwood et al., 2016).

### Qualitative data

#### Diagnostic background

Participants were invited to describe their diagnostic journey during the semi-structured interview. Because the interview prioritized participant-led discussion, individuals were not required to provide detailed clinical histories and could choose how much information to share. Several participants briefly described the timing of their diagnosis; for example, Participant 5 reported receiving a diagnosis during childhood (around age 10), whereas Participant 3 described receiving a diagnosis later in adolescence (age 18). Other participants referred more generally to having been diagnosed previously but did not elaborate on the assessment process.

#### Onboarding and theBlu

Participant 1 was the only participant who reported having an Oculus Quest (VR Headset) at home. They frequently used it for gaming purposes, and this was evident as they referred to “theBlu” series (WEVR, 2016) as an “underwater combat” and challenged themselves to try the different episodes (reef migration, whale encounter, and luminous abyss). All participants enjoyed the face-to-face encounter with different oceanic animals and Participant 2 took a particular interest in the sea urchins:

… it just makes me want to like stroke them. I’ve never experienced anything like this before… I like the squidginess here…

Similarly, participant 5 expressed a desire to physically interact with their environment: “you kind of want to jump?.” They then proceeded to sit down on the floor as they expressed a greater interest in watching the movements of the fish from that angle. Participant 3 also found the movements of the fish very relaxing: “I like them. The fact that they are bobbing up and down. The fish are very fluid motion.”

“TheBlu” (WEVR, 2016) features offered a sense of calmness to most participants and initiated conversations about their own personal experiences. For instance, Participant 1’s encounter with the jellyfish initiated a story about holidays: “whoa, well these things need to back off? I have been stung three separate times” whilst Participant 2 shared their hatred toward sand: “No, it feels like really claustrophobic. With all the sand in between the crevices of my toes, it feels like I can’t escape. Like I want to just take my skin off.”

#### Free drawing with OpenBrush: case studies

The preference for drawing in bright colors against a darker environment, such as the night sky or space, was the most common feature. Most participants drew similarly to what they would draw on paper and tended to pick their favorite colors. Participants frequently used the teleport feature and enjoyed seeing their drawings from a different angle.

#### Case 1: participant 1, 24, male

**FIGURE 1:**
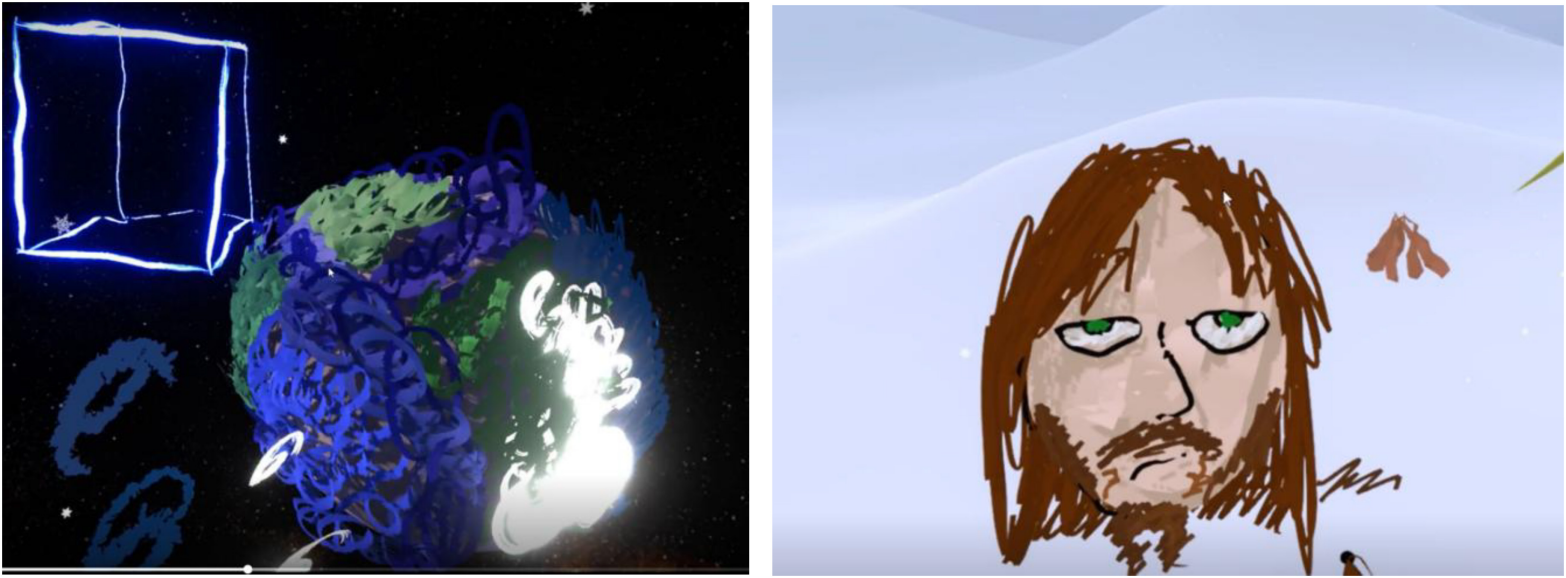
Example drawings produced by participant 1.

Participant 1 was fascinated by the space environment and spent a significant amount of time re-creating the Earth. They added specific details to differentiate between the various aspects of their drawings and used colors commonly associated with them, such as green for land areas, blue for water areas, and brown for wooded areas. Participant 1 drew in circular motions and enjoyed exploring the various brush features, particularly the fire brush (Figure 1).

Participant 1 did not consider themselves artistically talented. However, they specified that daydreaming and drawing people was something they often did in school, and they enjoyed the freedom of VR; “…Yeah. I love this. This is so cool. It’s my little canvas…”

Their attention to detail was evident in the self-portrait, which included specific details such as facial hair, eye color, and skin tone. Participant 1 found drawing 3D shapes difficult and expressed a stronger interest in cartoon character drawing.

Objects are… they’re geometric?. There is a specific way to draw a chair, you know, you can kind of make it look different, but it has to have certain dimensions. I can never get the dimensions done perfectly… but a person, a person can have wild, uh, dimensions and geometry … It’s very organic designs. So, organic designs can look sort of imperfect, like this and still look fairly okay…

#### Case 2: participant 2, 19, male

**FIGURE 2:**
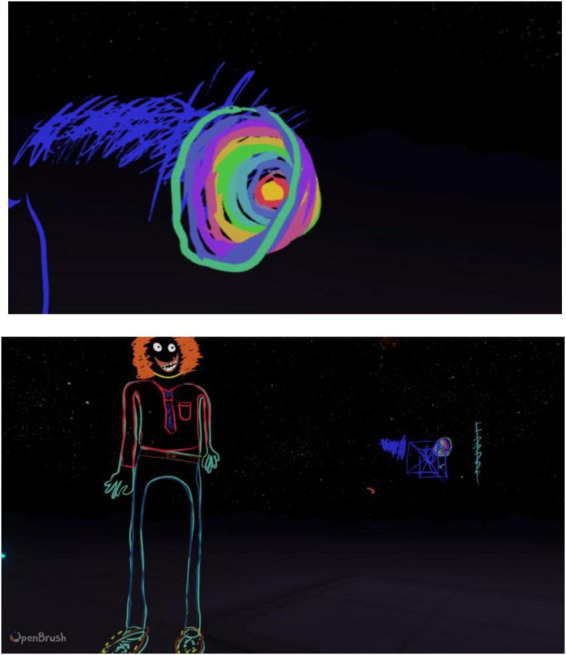
Example drawings produced by participant 2.

Participant 2 was the only participant who disclosed having another diagnosis and described how their synesthesia and autism can simultaneously influence their perceptual experiences (Figure 2). Almost immediately, Participant 2 created a drawing they used to do as a child:

“… I like the, I don’t know, the softness in a way. Like the circle is very soft… I like squares because they all fit into each other. But, with circles… like creatively, they are very satisfying because you don’t really get circles in, in our lives very much… Like in the supermarket. I don’t like the corners. Like where the price tags are on the shelf comes out a little bit and so jaggy. I don’t like that…”

Although participant 2 selected vibrant and neon colors in VR, this was different to reality as colors are something they constantly seek. Therefore, they try to avoid the stimulation where possible. Contrary to other participants, participant 2 did not enjoy the movements of the stars feature:

“… it’s a bit too much, really, um, yeah. I much prefer something a bit more still, and in my own control … I don’t like stuff out of my control…”

They included fine details in the drawing of a person, drawing the outline of the person in neon colors to add a futuristic aspect. Participant 2 did not assign any gender to their person, stating that he “would like them to be happy” and proceeded to add a hairclip to the person’s hair. They expressed an interest in cartoon characters and made sure to include shoelaces, a tie and a shirt pocket:

“…It always annoys me when cartoons don’t have shoelaces…I quite like this actually because the blue is like his skin and t-shirts never go on straight anyways. That’s another thing that cartoons get wrong…”

Participant 2 was very comfortable in discussing their synesthetic experiences and gave amazing insight into how it all works as they were able to draw an A-note, based purely on synesthetic associations.

#### Case 3: participant 3, 26, female

**FIGURE 3:**
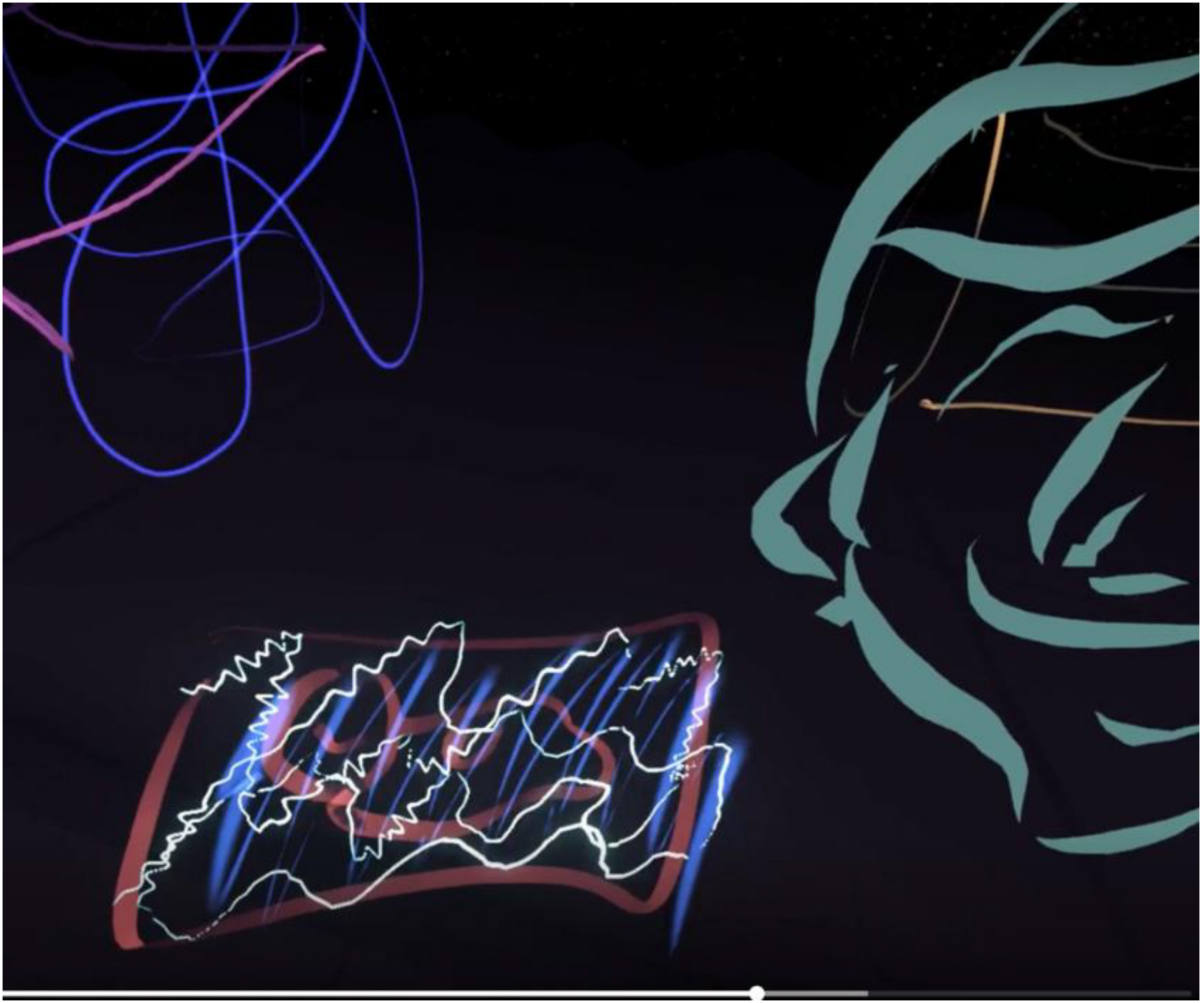
Example drawings produced by participant 3.

Out of all the participants, participant 3 was the most drawn to the animated brush features such as the stars, embers, and the neon lights. They continuously used them in the drawings, describing themselves as a “magpie” in daily life as they are drawn to anything bright and shiny. In VR they became particularly interested in the neon colors (Figure 3).

Participant 3 experimented with all of the brush features, and when recreating a dream, they used the waveform feature to depict a baby scan and the color blue to reflect negative emotions. Participant 3 used the entire space when drawing and had a spiraling drawing style similar to other participants. Their drawings were bright and busy, and they found it easier to express themselves in VR.

“…Well, I can, I can just draw. Especially in this environment, I can be myself… Like this actually gets erased, whereas it doesn’t on paper, I’m just wasting paper.”

#### Case 4: participant 4, 27, male

**FIGURE 4:**
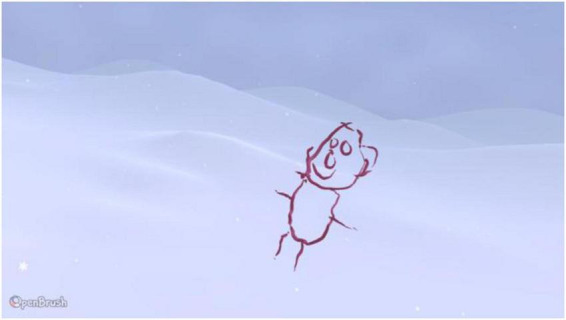
Example drawings produced by Participant 4.

Participant 4 was the only participant who drew in the snow environment, claiming that the brightness was more appealing to them. Participant 4 expressed some difficulty drawing in 3D and did not really grasp the 360 interactive aspect of VR out of all the participants who drew a cube in the study. They chose an ink brush for the self-portrait, which was simply drawn with a stickman body (Figure 4).

Notably, Participant 4’s movements in VR were restricted and they kept their hands close to the body, making it difficult to navigate the two controllers. Because the OpenBrush package requires the participant to be able to extend their arms quite easily, Participant 4 preferred *theBlu* series (WEVR, 2016).

#### Case 5: participant 5, 20, female

Because Participant 5 had a physical disability that affected their right side, they completed the drawing task with their left hand (different to the other participants). This change was simple to make in VR and had no effect on the study’s design.

Participant 5 mentioned before the study that they draw and doodle a lot, which was reflected in their drawings. They expressed difficulty drawing in depth and expressed no desire to draw a cube. All of their character drawings, like those of others, were in 2D. They enjoyed drawing animals in general, but cats in particular because of their simplicity:

“… Because cats you can just put sharpy ears on top of them. But dogs they are very different because they’re different. They’re different breeds of dogs. There are different types of coloring and fur, and ear shapes, but with eh, cats, it’s very simplistic…”

**FIGURE 5:**
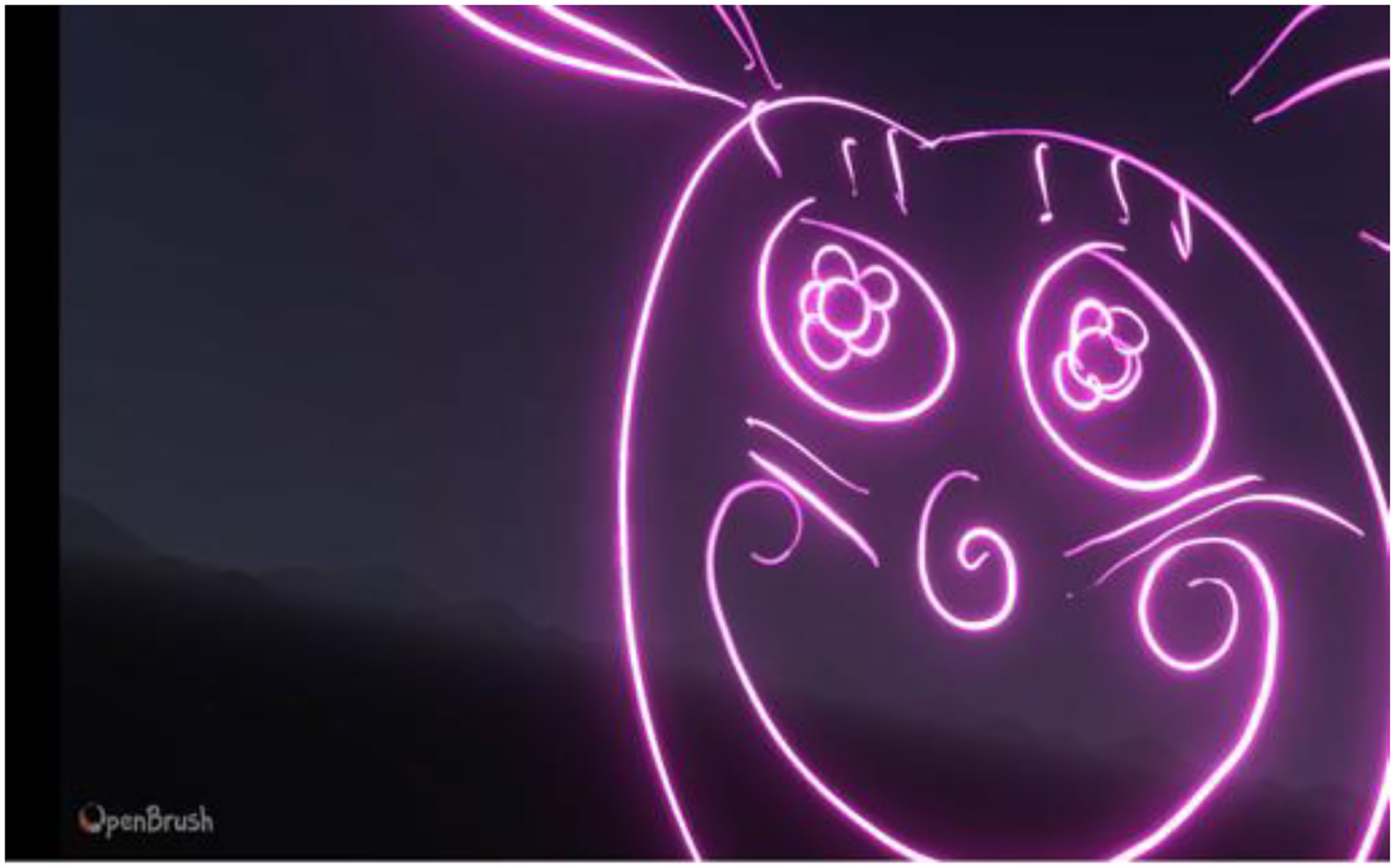
Example drawings produced by participant 5.

While they may consider their drawings to be simplistic, their drawings of cats were vibrant and varied in shape and size (Figure 5). Participant 5 used bright pinks and purples against a dark background, and they added unusual details like flowers in place of the cats’ eyes.

They drew in a spiraling pattern and filled the entire room with their drawings. Participant 5, like the other participants, proceeded to draw something they are familiar with. Notably, Participant 5 did indicate that although the VR was relatively simple to use, the effort that was required was quite strenuous:

“… If you could make it a bit more accessible just to people because it takes a lot to control this… No, it’s just, it’s just, it’s hard. This is the personal me, just because of my [diagnosed medical condition], but I think anybody could adapt to it…”

#### Case 6: participant 6, male

Although Participant 6 had never used VR before, they could relate it to the film *Ready Player One* (https://www.imdb.com/title/tt1677720/). This was beneficial to their understanding of the technology. Participant 6, like the other participants, drew in VR what they liked to draw on paper. Their special interest was super-heroes, and they were constantly testing colors and designs on the side of the drawing to ensure they were just right:

“…I’m going to need something about maybe, maybe not a little bit of. Hmm, I want to do a little bit of eyes. … I was going to do something about from the eyes, maybe a like wearing goggles and stuff…”

**FIGURE 6:**
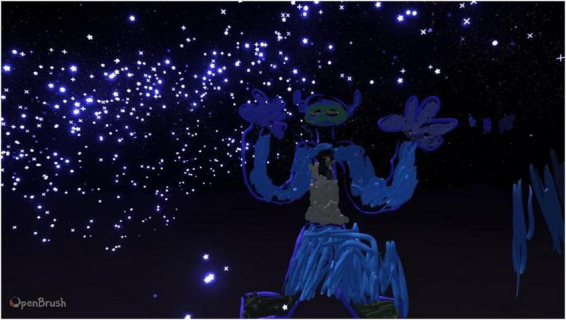
Example drawings produced by participant 6.

They referred to their super-hero as “T-man” and it soon became apparent that this reflected an important area of Participant 6’s life (Figure 6):

“…T word is because he’s a titanium man…Superhero. Because, because I can feel the metals inside of me because I did have to, I did have to get my operation …”

They went on to describe the super-physical hero’s strength, adding muscular features to the drawing. Participant 6 took a few moments at the end of the study to reflect on the surroundings:

“…I just enjoyed watching for the night sky…But before I lift up the headset. because I found myself very peaceful…”

### Thematic analysis: interaction with the virtual worlds (Theme 1)

To evaluate the suitability of VR for autistic participants, we examined their experiences during the OpenBrush drawing task and follow-up debrief discussions. Three overarching themes emerged: Immersion, Artistic Expression and Individuality, and Escapism.

### Immersion

The immersive nature of the 360-degree VR space was consistently appreciated. Participants described how moving within the environment enhanced their experience and understanding of space and scale. One participant shared:

“…I like being able to walk around it? Very cool… It helps a lot to have 3D space, so I can actually see within the space, that it’s supposed to be perceived in instead of having to throw a 3D image in a 2D space…”

This ability to explore the artwork from multiple perspectives contributed to a sense of presence and engagement. VR also enabled access to novel, imaginative environments. For example, Participant 2 reflected:

“…Look, you never get presented with things like that in real life. I love it…”

Such comments illustrate how VR’s immersive capacity can create compelling, accessible experiences for individuals who may find real-world environments challenging or overstimulating.

### Artistic expression and individuality

This theme revealed diverse and deeply personal relationships with creativity. All participants used their drawings to express identity and interests, although in very different ways. While Participant 2 voiced discomfort with open-ended creative tasks:

“… I find it hard to be creative. It’s one of the things I have always like really struggled with because I like things the way they are. If that makes sense. Like, I’m not very good at thinking outside of the box, the box is nice, why leave it?.”

Others found drawing in VR to be a therapeutic and grounding activity. Participant 3 described:

“…it calms me, I have done it since I was a wee girl…”

In contrast, Participant 5 emphasized the simple enjoyment of art:

“I don’t really like when people say does it express, I don’t really understand words like that, I, I just like to do it. There’s no such thing as expresses who I am, no, I just like it.”

These reflections highlight that creativity need not be symbolic or deeply conceptual-it may instead serve as a stabilizing and joyful form of self-expression.

### Escapism

VR offered a safe space to retreat, experiment, and exert control over one’s surroundings-something often difficult in the real world. Participant 3 noted the freedom to undo or reset changes:

“. like, I didn’t like the snow, so I could easily say, no, no, no. Return to the beginning.”

Participant 2 also valued the controlled exposure VR could offer:

“… So for example, like a bus station, um, it would give me different stimulus. Stimuli, but I know I can leave if I wanted to and I could play it’s all in my control. Whereas, you know, in the real world, it’s not, and that’s the worst part about it, really?.”

They later added:

“stay in there for hours. Given the opportunity. I would just sit there alone…”

The VR environment’s calming nature resonated widely. Participant 3 described the tranquil effect of the night sky:

“. I’d just love the stars, go into the night and just look at the stars and relax.”

Similarly, Participant 6 commented on the peace enabled by the reduced auditory input.

### Thematic analysis: daily experiences (Theme 2)

To further understand participants’ perspectives on autism and perception, we analyzed their responses to the debrief questions. Three themes were identified: Situational Adjustment, Autistic Perceptual Differences, and Sharing the Autistic Perspective through VR.

### Masking and situational adjustment

Participants had diverse diagnostic journeys, with some noting delays in support. Participant 5, diagnosed at age 10, recalled:

“…lack of understanding. Lack of caring…”

Participant 3, diagnosed at 18, shared how stigma influenced their choice to keep their diagnosis private:

“… I feel as if I have been judged a lot more cause people see autism as a disability. Like something to be disgraced with… if you don’t look as if you have autism then you are treated differently than people that do look like they have autism…”

Several participants described how they learned to mask autistic traits. Participant 2 explained:

“…they would teach me, you know, how to stand, you know, just normally, and rather than slouching up and curling up in a ball on the floor, you know… and before you know it, I can mask better than most Oscar winners could act…”

Participant 5 highlighted how their home environment supported desensitization to overwhelming stimuli and rapid situational adjustments.

### Autistic sensory differences affect perception

Participants reported highly individualized sensory responses. Participant 2 explained:

“I could be having sensory overload in the same environment as someone else’s perfectly fine, but we could both be autistic.”

Participant 5 noted:

“it’s more like the IKEA lights, when you go to IKEA you see those lights.”

Others struggled to differentiate their experience from others’. Participant 3 said:

“everyone’s different”

While Participant 1 reflected:

“… I’ve never, I’ve never had any experience where I thought to myself, I would’ve, I would’ve done this differently if I didn’t have autism like I couldn’t point to a part of my personality and go that’s definitely autism…”

These observations reflect the heterogeneity of autistic sensory and perceptual experience.

### Sharing the autistic perspective through VR

Participants were largely enthusiastic about the potential of VR to help others better understand autistic perception. Participant 3 suggested family members might benefit from trying it, while Participant 2 expressed:

“… I can’t ever like tell people what sensory overload is like. You know, VR, could help do that… it would be a way to try and bridge that gap between neurotypical perception and neurodiverse perception. But for sure, it’s a very good tool…”

However, concerns about overstimulation were raised:

“… it is actually really quite hard to focus in on those, those particular things that might send you a bit crazy, uh, that would cause sensory overload. But I think given enough time and enough implementation. It would be fantastic…”

Participant 5 questioned the true impact of such tools:

“. I don’t really think you can understand autism to an extent.”

And added:

“…Do the neurotypicals want to come meet us, or do you want us to jump into your own border… like jump into your world? Cause that’s not going to do any good.”

### Drawings

Participants were free to choose their VR environment. Most selected darker backgrounds (e.g., night sky, space) and similar colors (blues, pinks, purples). Some (Participants 3 and 6) incorporated animated brushes like stars and embers.

**FIGURE 7:**
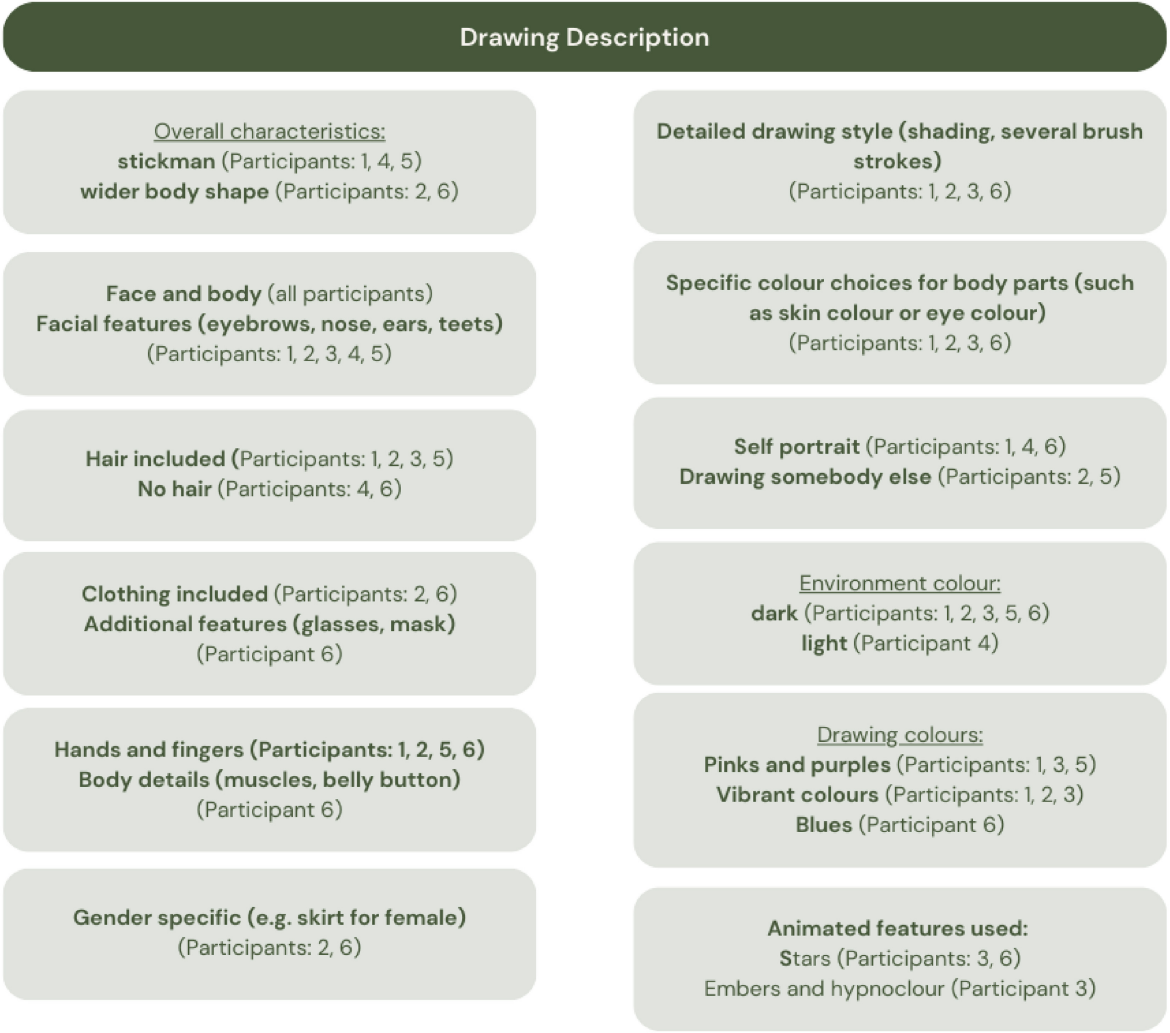
Summary of the characteristics of each drawing, environment and brushes used by individual participants.

Figure 7 summarizes brush choices and environmental preferences. Although no universal analysis method exists for free VR drawings, our observations suggest that those with higher AQ scores (Participants 2 and 6) tended to include more intricate features like facial expressions, hair, and layered brushwork. Others opted for simplified figures.

Visual material was analyzed using a descriptive visual–thematic approach. Drawings were examined for recurring features including color use, spatial organization, level of detail, subject matter, and brush type. These features were coded alongside verbal Think-Aloud and interview data, allowing triangulation between what participants said and what they produced visually.

These drawing styles may reflect differences in imagination, attention to detail, or familiarity with digital art tools-and will be explored further in future research.

## Discussion

This study aimed to explore the perceptual experiences of autistic individuals using an immersive, interactive environment in Virtual Reality (VR). By incorporating OpenBrush-a VR-based 3D drawing tool-we sought to provide a platform for self-expression and reflection on perception and creativity. Due to the novel design of the study, we also examined the feasibility of using OpenBrush in experimental settings with autistic participants. While participants’ verbal reports served as the primary data source, we also incorporated two established measures-the Autism Spectrum Quotient (AQ) and the Glasgow Sensory Questionnaire (GSQ)-to provide supplementary insights. Reflexive thematic analysis was used to interpret participants’ verbal and visual outputs. To our knowledge, this is the first study to employ VR drawing to investigate perceptual differences in autistic individuals.

### Quantitative data

Three participants (1, 4, and 6) had total GSQ scores that indicated a relatively low frequency of hyper- and hypo-sensitivity. These scores were even below the UK average of 46 (Millington et al., 2022), highlighting the heterogeneity of autism. Participant 2, by contrast, had a significantly elevated GSQ score—particularly in the visual, tactile, auditory, and olfactory subscales. Literature suggests that synesthesia may enhance some autistic traits (Ward et al., 2017), and in our study, Participant 2-who disclosed a synesthetic experience-scored a GSQ total of 120, well above the population mean.

Most participants showed elevated GSQ hypersensitivity subscale scores in comparison to their hypo-sensitivity scores. Previous research has reported positive associations between AQ and GSQ (Robertson and Simmons, 2013). We did not conduct correlation analyses in the present small sample, and therefore these measures are interpreted descriptively.

Most participants showed their highest scores on the AQ attention-switching subscale, which measures difficulty in shifting focus between stimuli (Baron-Cohen, 2001). This resonates with research by Reed and McCarthy (2012), who found that autistic children show reduced cross-modal attention switching. Given that VR involves multi-sensory input (visual, auditory, tactile), this factor should be considered in future research design. Self-report on imagination, attention, and sensory experience sometimes paralleled AQ subscale patterns, but given the exploratory nature and small sample, these relationships are described illustratively rather than inferentially.

The AQ scores in the present sample showed substantial variability, with several participants scoring close to or below commonly used cut-off points. Although the AQ is widely used as a screening tool, previous research has highlighted limitations in its sensitivity when applied to clinically diagnosed adults. For example, Ashwood et al. (2016) found that the AQ can yield substantial false negatives in referral samples, indicating that some individuals with confirmed autism diagnoses score below commonly used thresholds. Similarly, validation studies have reported variability in AQ performance across populations and contexts, highlighting that screening cut-offs such as 26 are not definitive indicators of diagnosis (Woodbury-Smith et al., 2005; Broadbent et al., 2013). More recent work has also emphasized that factors such as camouflaging, gender differences in presentation, and heterogeneity in autistic traits may influence AQ scores and contribute to under-identification in some groups (Belcher et al., 2023; Perry et al., 2021; Bradley et al., 2021; Ferraiolo et al., 2026). In light of these findings, the AQ scores in the present study are interpreted descriptively to illustrate variability in self-reported traits rather than as indicators of diagnostic validity. The discrepancy between expected AQ distributions and observed scores therefore reflects known psychometric limitations of the instrument rather than evidence regarding participants’ diagnostic status.

### Qualitative data

**FIGURE 8:**
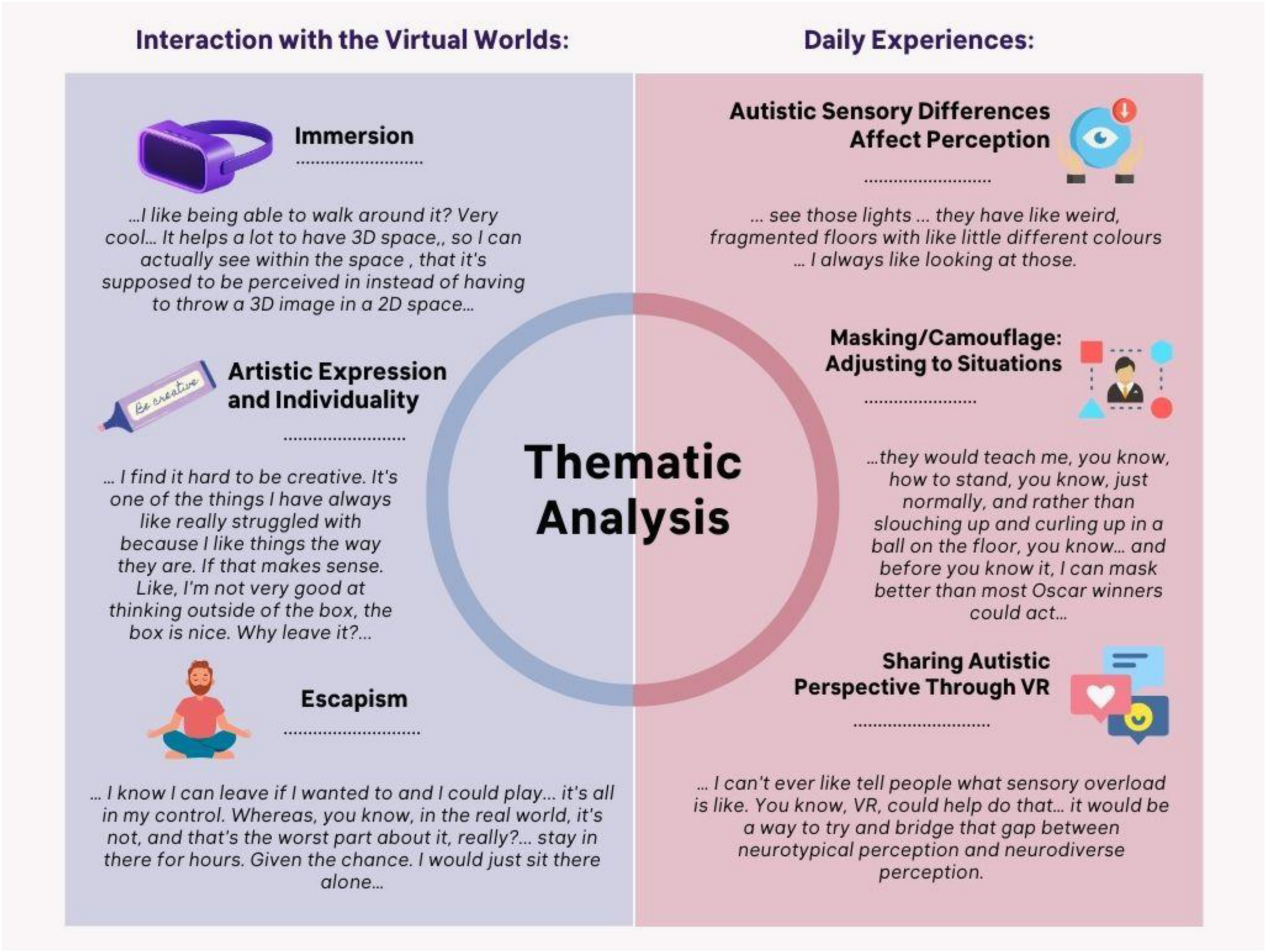
Summary of thematic analysis conducted, and subthemes identified.

Our findings reinforce several insights already present in the literature, particularly around environmental control. Prior studies have reported that the ability to manage sensory input can reduce stress in autistic individuals (Robertson and Simmons, 2015). Our participants also highlighted the importance of being able to modify their VR surroundings. This sense of control may have enabled them to explore OpenBrush features they would usually avoid in real life-for example, vibrant colors (as seen with Participant 2; Figure 8).

### OpenBrush drawings: case studies

The visual analysis of drawings captured participants’ creative preferences and perceptual styles. Many preferred using bright, contrasting colors against dark backgrounds. Drawing approaches varied, reflecting a range of cognitive and aesthetic inclinations.

Participant 1, for instance, created a highly detailed self-portrait with accurate colors for skin tone, eyes, and facial hair. Although such precision is often associated with a low imaginative profile in the literature (Scott and Baron-Cohen, 1996), this contrasted with their AQ-Imagination score. Participants 2 and 6 drew imaginative characters with detailed features such as goggles and shoelaces, consistent with a detail-oriented cognitive style (Darewych et al., 2018).

Participants 3, 4, and 5 showed less interest in drawing human figures. This may reflect a broader trend in autistic expressive art, where preference is often given to non-social subjects (Schweizer et al., 2014; Darewych et al., 2018). However, this does not necessarily suggest limited imagination. For instance, Participant 5’s use of floral elements for eyes in cat drawings reflected originality and creativity.

Imaginative responses in drawing tasks may also depend on the prompt’s social content. Ten Eycke and Müller (2015) found that autistic children showed more imaginative output when asked to draw a house than a person. Thus, future studies should carefully consider task framing.

Despite the 3D nature of VR, most participants struggled with depth perception, drawing primarily in 2D. This aligns with findings in art therapy studies suggesting that depth perception can be challenging for some autistic individuals (Alter-Muri, 2017; Vaisvaser, 2019). The preference for familiar drawings may reflect autistic characteristics like insistence on sameness. Importantly, this tendency could also be present in neurotypical participants using unfamiliar tools like VR for the first time and should be investigated further with control groups.

None of the participants reported motion sickness or dizziness (Riva and Serino, 2020), although some required time to adjust to the lighting in VR-corresponding with their GSQ-visual scores. Notably, OpenBrush allowed users to control their sensory environment, an essential feature previously identified as beneficial for autistic individuals (MacLennan et al., 2021; Robertson and Simmons, 2015). The ability to erase drawings encouraged creative experimentation.

Participant 2 described their VR drawings as a “little palace,” highlighting the sense of ownership and immersion the tool provided. VR offered a way to disconnect from real-world stressors-paralleling research on escapism and gaming in autistic individuals (Engelhardt et al., 2017). Participants frequently lost track of time and were reluctant to leave the VR space. This was not problematic in our setting but should be monitored in extended use cases. These immersive and expressive qualities suggest significant potential for therapeutic applications.

Participants reported varied sensory sensitivities. Most described hyper-reactivity, although Participant 5-who reported few sensory challenges-scored higher on GSQ hypo-sensitivity. Participants also described sensory events as physically uncomfortable, consistent with previous studies (Robertson and Simmons, 2015; Robertson et al., 2012). Several noted that emotional state, fatigue, and uncertainty impacted their sensory processing, which may explain some of the intra-individual variability reported here. These factors should be formally measured in future research.

Participant 2’s reference to fragmented perception in supermarkets echoes descriptions of perceptual fragmentation in autism (Kanner, 1943; Booth and Happé, 2018). Such qualitative insights may reveal aspects of perception that cannot be captured using questionnaires alone.

### Limitations and future considerations

There are several limitations to this study. First, the small sample size, whilst acceptable in qualitative studies, (*N* = 6) limits generalizability. Diagnoses were self-reported and not independently verified. Though the AQ and GSQ helped contextualize participants’ profiles, our primary goal was exploratory, and the emphasis was on qualitative data. AQ scores showed substantial heterogeneity and should not be interpreted as diagnostic or severity indicators. AQ scores showed an anomalous distribution in this diagnosed sample, with several participants scoring close to or below typical cut-offs despite self-reported formal diagnoses, consistent with known limitations of the AQ in adult autistic populations. Due to technical issues, full VR session recordings were not available for two participants, resulting in inconsistent data volumes.

Second, no control group was included, which prevents comparison of perceptual experiences between autistic and neurotypical individuals. Participants also indicated that emotional state and stress levels influenced their perception, but these were not systematically recorded.

Third, each participant received one VR session lasting approximately an hour. For those unfamiliar with VR, this may not have been enough time to fully adapt. Prior research on art therapy suggests that repeated exposure may enhance expressive capacity over time (Schweizer et al., 2014). Future studies could incorporate multiple sessions and longitudinal observation.

Despite these limitations, the study demonstrates the potential of VR as a tool for exploring and understanding perceptual differences. It offers a supportive and flexible platform for autistic self-expression. These findings suggest VR could be particularly effective for therapeutic use and as a communication bridge between autistic and non-autistic people (Bogdashina, 2016; Savickaite et al., 2021).

Participants self-reported having received a formal autism diagnosis, and diagnostic documentation was not independently verified. As a result, the possibility of misclassification bias cannot be fully excluded. However, the study adopted a phenomenological and neurodiversity-affirming approach that prioritized participants’ subjective experiences rather than clinical categorization. Participants were invited to describe their diagnostic journeys but were not required to provide detailed clinical information, as the interview format allowed participants to guide the discussion toward aspects of their experiences they considered most relevant. This approach aligns with qualitative research traditions that aim to minimize power imbalances between researchers and neurodivergent participants and to respect participants’ autonomy in discussing their medical histories.

## Conclusion

Understanding perception in autism requires a first-person, participant-led approach. This study presented a novel use of VR-via the OpenBrush drawing tool-to investigate autistic individuals’ perceptual worlds. Combining visual, auditory, and narrative data enabled a rich exploration of personal experience. VR was well-received by all participants, who described it as immersive, calming, and creatively liberating.

These findings support the view that VR can serve both research and therapeutic purposes. Future work should build on this foundation, integrating larger and more diverse samples, repeated sessions, and neurotypical comparison groups to deepen understanding of perceptual experiences in autism.

## Data Availability

The datasets presented in this article are not readily available because of limitations in the ethical approval. Requests to access the datasets should be directed to s.savickaite@exeter.ac.uk.

## References

[B1] Alter-Muri S. (2017). Art education and art therapy strategies for autism spectrum disorder students. *Art Educ.* 70, 20–25. 10.1080/00043125.2017.1335536

[B2] American Psychiatric Association [APA]. (2022). *Diagnostic and Statistical Manual of Mental Disorders*, 5th Edn. Washington, DC: American Psychiatric Publishing.

[B3] Arsal G. Eccles D. W. (2017). The think aloud method: What is it and how do I use it? *Qual. Res. Sport Exerc. Health* 9 514–531. 10.1080/2159676X.2017.1331501

[B4] Ashwood K. L. Gillan N. Horder J. Hayward H. Woodhouse E. McEwen F. S. et al.. (2016). Predicting the diagnosis of autism in adults using the Autism-spectrum quotient (AQ) questionnaire. *Psychol. Med.* 46 2595–2604. 10.1017/S0033291716001082 27353452 PMC4988267

[B5] Baron-Cohen S. (2001). Theory of mind in normal development and autism. *Prisme* 34, 174–183.

[B6] Baron-Cohen S. Wheelwright S. Skinner R. Martin J. Clubley E. (2001). The Autism-spectrum quotient (AQ): Evidence from Asperger syndrome/high-functioning autism, males and females, scientists and mathematicians. *J. Autism Dev. Disord.* 31 5–17. 10.1023/A:1005653411471 11439754

[B7] Belcher H. L. Morein-Zamir S. Stagg S. D. Ford T. J. (2023). Gender bias in autism screening: Measurement invariance of different model frameworks of the Autism Spectrum Quotient. *BJPsych Open* 9:e79. 10.1192/bjo.2023.70 37781848 PMC10594186

[B8] Bogdashina O. (2016). *Sensory Perceptual Issues in Autism and Asperger Syndrome: Different Sensory Experiences—Different Perceptual Worlds*, 2nd Edn. Philadelphia, PA: Jessica Kingsley Publishers

[B9] Booth R. D. Happé F. G. (2018). Evidence of reduced global processing in autism spectrum disorder. *J. Autism Dev. Disord.* 48 1397–1408. 10.1007/s10803-016-2724-6 26864159 PMC5861162

[B10] Botha M. Hanlon J. Williams G. L. (2021). Does language matter? Identity-first versus person-first language use in autism research: A response to Vivanti. *J. Autism Dev. Disord.* 51 870–878. 10.1007/s10803-020-04401-9 PMC7817071 33474662

[B11] Bradley L. Shaw R. Baron-Cohen S. Cassidy S. (2021). Autistic adults’ experiences of camouflaging and its perceived impact on mental health. *Autism Adulth.* 3 320–331. 10.1089/aut.2020.0071 36601637 PMC8992917

[B12] Braun V. Clarke V. (2006). Using thematic analysis in psychology. *Qual. Res. Psychol.* 3 77–101. 10.1191/1478088706qp063oa

[B13] Broadbent J. Galic I. Stokes M. A. (2013). Validation of Autism spectrum quotient adult version in an Australian sample. *Autism Res. Treatment* 2013:984205. 10.1155/2013/984205 23762552 PMC3665170

[B14] Cage E. Di Monaco J. Newell V. (2018). Experiences of autism acceptance and mental health in autistic adults. *J. Autism Dev. Disord.* 48 473–484. 10.1007/s10803-017-3342-7 29071566 PMC5807490

[B15] Chung S. J. Choi I. Han Y. Jo H. J. (2020). User-centred design for multi-user virtual reality art therapy (VRAT) system. *J. HCI Soc. Korea* 15 27–35. 10.17210/jhsk.2020.12.15.4.27

[B16] Darewych O. H. Newton N. J. Farrugie K. W. (2018). Investigating imagination in adults with autism spectrum disorder with art. Based assessments. *J. Dev. Disabil.* 23 27–36.

[B17] Engelhardt C. R. Mazurek M. O. Hilgard J. (2017). Pathological game use in adults with and without Autism spectrum disorder. *PeerJ* 5:e3393. 10.7717/peerj.3393 28663933 PMC5488854

[B18] English M. C. W. Gignac G. E. Visser T. A. W. Whitehouse A. J. O. Maybery M. T. (2020). A comprehensive psychometric analysis of autism-spectrum quotient factor models using two large samples: Model recommendations and the influence of divergent traits on total-scale scores. *Autism Res.* 13 45–60. 10.1002/aur.2210 31464106

[B19] Ferraiolo J. Rouse H. L. Pugliese C. E. (2026). Self-reported strengths and difficulties by autistic young adults. *J. Autism Dev. Disord* 10.1007/s10803-026-07260-0 41706308

[B20] Freeseman L. J. Colombo J. Coldren J. T. (1993). Individual differences in infant visual attention: Four-month-olds’ discrimination and generalization of global and local stimulus properties. *Cogn. Dev.* 8 419–435. 10.1111/j.1467-8624.1993.tb04195.x 8404264

[B21] Goldstein E. B. Brockmole J. R. (2016). *Sensation and Perception*, 10th Edn. Boston, MA: Cengage Learning.

[B22] Grandin T. (2006). *Thinking in Pictures, Expanded Edition: My Life With Autism.* New York, NY: Knopf Doubleday Publishing Group.

[B23] Guillemin M. (2004). Understanding illness: Using drawings as a research method. *Qual. Health Res.* 14 272–289. 10.1177/1049732303260445 14768462

[B24] Happé F. Frith U. (2020). Annual Research Review: Looking back to look forward—changes in the concept of autism and implications for future research. *J. Child Psychol. Psychiatry* 61 218–232. 10.1111/jcpp.13176 31994188

[B25] Higashida N. (2013). *The Reason I Jump: The Inner Voice of a Thirteen-Year-Old Boy with Autism*, eds Yoshida K. A. Mitchell D., Trans. (New York, NY: Random House).

[B26] Hoekstra R. A. Bartels M. Cath D. C. Boomsma D. I. (2008). Factor structure, reliability and criterion validity of the Autism-spectrum quotient (AQ): A study in Dutch population and patient groups. *J. Autism Dev. Disord.* 38 1555–1566. 10.1007/s10803-008-0538-x 18302013 PMC2516538

[B27] Horder J. Wilson C. E. Mendez M. A. Murphy D. G. (2014). Autistic traits and abnormal sensory experiences in adults. *J. Autism Dev. Disord.* 44 1461–1469. 10.1007/s10803-013-2012-7 24305777 PMC4022987

[B28] Icosa Gallery. (2021). *Open Brush [Virtual reality application].* Available online at: https://icosa.gallery/

[B29] Kaimal G. Carroll-Haskins K. Berberian M. Dougherty A. Carlton N. Ramakrishnan A. (2020). Virtual reality in art therapy: A pilot qualitative study of the novel medium and implications for practice. *Art Therapy* 37 16–24. 10.1080/07421656.2019.1659662

[B30] Kanner L. (1943). Autistic disturbances of affective contact. *Nervous Child* 2 217–250. 4880460

[B31] Lorenzo G. Gómez-Puerta M. Arráez-Vera G. Lorenzo-Lledó A. (2019). Preliminary study of augmented reality as an instrument for improvement of social skills in children with autism spectrum disorder. *Educ. Information Technol.* 24 181–204. 10.1007/s10639-018-9768-5

[B32] MacLennan K. Rossow T. Tavassoli T. (2021). The relationship between sensory reactivity, intolerance of uncertainty and anxiety subtypes in preschool-age autistic children. *Autism* 25 2305–2316. 10.1177/13623613211016110 34030518

[B33] Maskey M. Lowry J. Rodgers J. McConachie H. Parr J. R. (2014). Reducing specific phobia/fear in young people with autism spectrum disorders (ASDs) through a virtual reality environment intervention. *PLoS One* 9:e100374. 10.1371/journal.pone.0100374 24987957 PMC4079659

[B34] Millington E. Simmons D. R. Cleland Woods H. (2022). Brief report: Investigating the motivations and autistic traits of video gamers. *J. Autism Dev. Disord.* 52, 1403–1407. 10.1007/s10803-021-04994-x 33886033

[B35] National Autistic Society. (2024). *Guidance for the Media: How to Talk and Write About Autism (Identity-First Language Guidance).* London: National Autistic Society

[B36] Nicolaidis C. Raymaker D. Kapp S. Baggs A. Ashkenazy E. McDonald K. et al.. (2019). The AASPIRE practice-based guidelines for the inclusion of autistic adults in research as co-researchers and study participants. *Autism* 23 2007–2019. 10.1177/1362361319830523 30939892 PMC6776684

[B37] Oster G. D. Crone P. G. (2004). *Using Drawings in Assessment and Therapy: A Guide for Mental Health Professionals.* Milton Park: Routledge.

[B38] Perry E. Mandy W. Hull L. (2021). Understanding camouflaging as a response to autism-related stigma: A social identity theory approach. *Autism* 25 800–810. 10.1177/1362361320961699 33788076 PMC8813820

[B39] QSR International. (2018). *NVivo (Version 12) [Computer software].* Available online at: https://lumivero.com/products/nvivo/

[B40] Rebenitsch L. Owen C. (2016). Review on cybersickness in applications and visual displays. *Virtual Reality* 20 101–125. 10.1007/s10055-016-0285-9

[B41] Reed P. McCarthy J. (2012). Cross-modal attention-switching is impaired in autism spectrum disorders. *J. Autism Dev. Disord.* 42 947–953. 10.1007/s10803-011-1324-8 21720723

[B42] Riva G. Serino S. (2020). Virtual reality in the assessment, understanding and treatment of mental health disorders. *J. Clin. Med.* 9:3434. 10.3390/jcm9113434 33114623 PMC7693021

[B43] Robertson A. E. Simmons D. R. (2013). The relationship between sensory sensitivity and autistic traits in the general population. *J. Autism Dev. Disord.* 43 775–784. 10.1007/s10803-012-1608-7 22832890

[B44] Robertson A. E. Simmons D. R. (2015). The sensory experiences of adults with autism spectrum disorder: A qualitative analysis. *Perception* 44 569–586. 10.1068/p7833 26422904

[B45] Robertson C. E. Baron-Cohen S. (2017). Sensory perception in autism. *Nat. Rev. Neurosci.* 18 671–684. 10.1038/nrn.2017.112 28951611

[B46] Robertson C. E. Martin A. Baker C. I. Baron-Cohen S. (2012). Atypical integration of motion signals in autism spectrum conditions. *PLoS One* 7:e48173. 10.1371/journal.pone.0048173 23185249 PMC3502435

[B47] RStudio Team. (2021). **RStudio [Computer software].* Available online at: https://posit.co/blog/introducing-rstudio-team/

[B48] Russell G. Kapp S. K. Elliott D. Elphick C. Gwernan-Jones R. Owens C. (2019). Mapping the autistic advantage from the accounts of adults diagnosed with autism: A qualitative study. *Autism Adulth.* 1 124–133. 10.1089/aut.2018.0035 31058260 PMC6493410

[B49] Ruzich E. Allison C. Smith P. Watson P. Auyeung B. Ring H. et al.. (2015). Measuring autistic traits in the general population: A systematic review of the Autism-Spectrum Quotient (AQ) in a nonclinical population. *Mol. Autism* 6:2. 10.1186/2040-2392-6-2 25874074 PMC4396128

[B50] Sadler-Smith E. (2011). *The Intuitive Mind: Profiting from the Power of Your Sixth Sense.* Hoboken, NJ: John Wiley & Sons.

[B51] Sapey-Triomphe L. A. Moulin A. Sonié S. Schmitz C. (2018). The glasgow sensory questionnaire: Validation of a French language version and refinement of sensory profiles of people with high autism-spectrum quotient. *J. Autism Dev. Disord.* 48 1549–1565. 10.1007/s10803-017-3422-8 29189917

[B52] Savickaite S. McNaughton K. Gaillard E. Amaya I. McDonnell N. Millington E. et al.. (2021). Using HMD Virtual Reality to investigate individual differences in visual processing styles. *PsyArXiv Preprints* 10.

[B53] Schweizer C. Knorth E. J. Spreen M. (2014). Art therapy with children with Autism spectrum disorders: A review of clinical case descriptions on ‘what works’. *Arts Psychotherapy* 41 577–593. 10.1016/j.aip.2014.10.009

[B54] Scott F. J. Baron-Cohen S. (1996). Imagining real and unreal things: Evidence of a dissociation in autism. *J. Cogn. Neurosci.* 8 371–382. 10.1162/jocn.1996.8.4.371 23971507

[B55] Simmons D. R. Todorova G. K. (2018). Local versus global processing in autism: Special section editorial. *J. Autism Dev. Disord.* 48 1338–1340. 10.1007/s10803-017-3452-2 29380272

[B56] Simmons D. R. Robertson A. E. McKay L. S. Toal E. McAleer P. Pollick F. E. (2009). Vision in autism spectrum disorders. *Vision Res.* 49 2705–2739. 10.1016/j.visres.2009.08.005 19682485

[B57] Sousa Santos B. Dias P. Pimentel A. Baggerman J. W. Ferreira C. Silva S. et al.. (2009). Head-mounted display versus desktop for 3D navigation in virtual reality: A user study. *Multimed. Tools Appl.* 41, 161–181. 10.1007/s11042-008-0223-2

[B58] Takayama Y. Hashimoto R. Tani M. Kanai C. Yamada T. Watanabe H. et al.. (2014). Standardization of the Japanese version of the Glasgow sensory questionnaire (GSQ). *Res. Autism Spectrum Disord.* 8 347–353. 10.1016/j.rasd.2013.12.017

[B59] Ten Eycke K. D. Müller U. (2015). Brief report: New evidence for a social-specific imagination deficit in children with autism spectrum disorder. *J. Autism Dev. Disord.* 45 213–220. 10.1007/s10803-014-2206-7 25103864

[B60] Thompson-Hodgetts S. Labonte C. Mazumder R. Phelan S. (2020). Helpful or harmful? A scoping review of perceptions and outcomes of autism diagnostic disclosure to others. *Res. Autism Spectrum Disord.* 77:101598. 10.1016/j.rasd.2020.101598

[B61] Vaisvaser S. (2019). Moving along and beyond the spectrum: Creative group therapy for children with autism. *Front. Psychol.* 10:417. 10.3389/fpsyg.2019.00417 30914987 PMC6423063

[B62] Wagemans J. Elder J. H. Kubovy M. Palmer S. E. Peterson M. A. Singh M. et al.. (2012). A century of Gestalt psychology in visual perception: I. Perceptual grouping and figure–ground organization. *Psychol. Bull.* 138 1172–1217. 10.1037/a0029333 22845751 PMC3482144

[B63] Ward J. Hoadley C. Hughes J. Smith P. Allison C. Baron-Cohen S. et al.. (2017). Atypical sensory sensitivity as a shared feature between synaesthesia and autism. *Sci. Rep.* 7:41155. 10.1038/srep41155 28266503 PMC5339734

[B64] WEVR. (2016). *theBlu [Virtual Reality Application].* Available online at: https://wevr.com/

[B65] Williams D. (1992). *Nobody Nowhere: The Extraordinary aUtobiography of An Autistic Girl.* New York, NY: Doubleday

[B66] Woodbury-Smith M. R. Robinson J. Wheelwright S. Baron-Cohen S. (2005). Screening adults for Asperger syndrome using the AQ: A preliminary study of its diagnostic validity in a clinical sample. *Psychol. Med.* 35 331–335. 10.1017/S0033291704003092 16119474

